# Complete chloroplast genome sequence of *Gigantochloa verticillata* (Bambusodae)

**DOI:** 10.1080/23802359.2019.1702484

**Published:** 2020-02-06

**Authors:** Yang-Yang Zhang, Li-Li Fan, Feng-Ying Zheng, Ting Zhao, Jun-Dong Rong, Li-Guang Chen, Yu-Shan Zheng

**Affiliations:** a College of Forestry, Fujian Agriculture and Forestry University, Fuzhou, China;; b Key Laboratory of Bamboo Institute at College of Forestry, Fuzhou, China

**Keywords:** *Gigantochloa verticillata*, plastid genome, phylogeny, Bambusodae

## Abstract

*Gigantochloa verticillata* is produced in Mengla and Jinghong, Yunnan Province, China, and cultivated in Hong Kong. Vietnam, Thailand, India, Indonesia, and Malaysia are distributed and cultivated. We determined the complete chloroplast genome sequence for *G. verticillata* using Illumina sequencing data. The complete chloroplast sequence is 139,489 bp, including large single-copy (LSC) region of 83,062 bp, small single-copy (SSC) region of 12,877 bp, and a pair of invert repeats (IR) regions of 21,775 bp. Plastid genome contain 132 genes, 85 protein-coding genes, 39 tRNA genes, and 8 rRNA genes. Phylogenetic analysis based on 23 chloroplast genomes indicates that *G. verticillata* is closely related to *Dendrocalamus latiflorus* in Bambusodae.

*Gigantochloa verticillata* belongs to the family Bambusodae, it is mainly produced in tropical monsoon forests with an altitude of 400–800 meters, which can form a mixed forest of bamboo and wood (Zhang 2013). Among the 18 kinds of ornamental bamboo species commonly found in gardens, the flowering giant bamboo has the highest green amount (three-dimensional green amount) and can effectively absorb SO_2_, Cl_2_ in the environment. It is one of the excellent ornamental bamboo species for purifying atmospheric SO_2_, Cl_2_ (Hong 2014). Qiu used the set pair analysis to comprehensively assess the ecological adaptability, ornamental and ecological benefits, and the results showed that the giant bamboo was an excellent cluster of bamboo species (Qiu 2005). In this study, we report the complete chloroplast genome (cp) of *G. verticillata* based on Illumina pair-end sequencing data.

The plant material of *G. verticillata* was collected from Fujian province, China (Fujian Agriculture and Forestry University Baizhuyuan, Fuzhou: 119°14′16″E, 26°5′7″N), and dried into silica gel immediately. The voucher specimen is kept at the Herbarium of College of Forestry, Fujian Agriculture and Forestry University (specimen code FAFU101301). DNA extraction from fresh leaf tissue, with 500 bp randomly interrupted by the Covaris ultrasonic breaker for library construction. The constructed library was sequenced PE150 by Illumina Hiseq Xten platform, approximately 2GB data generated. Illumina data were filtered by script in the cluster (default parameter: -L 5, -p 0.5, -N 0.1). Complete plastid genome of *Arundinaria faberi* (GeneBank accession: JX513414) as reference, plastid genome of *G. verticillata* was assembled by GetOrganelle pipe-line (https://github.com/Kinggerm/GetOrganelle), it can get the plastid-like reads, and the reads were viewed and edited by Bandage (Wick et al. 2015). Assembled chloroplast genome annotation base on comparison with *A. faberi* by Geneious v 11.1.5 (Biomatters Ltd, Auckland, New Zealand) (Kearse et al. 2012). The annotation result was drawn with the online tool OGDRAW (http://ogdraw.mpimp-golm.mpg.de/) (Lohse et al. 2013).

The complete plastid genome sequence of *G. verticillata* (GenBank accession: MN688203) was 139,489 bp in length, with a large single-copy (LSC) region of 83,062 bp, a small single-copy (SSC) region of 12,877 bp, and a pair of inverted repeats (IR) regions of 21,775 bp. Complete chloroplastid genome contains 132 genes, there were 85 protein-coding genes, 39 tRNA genes, and 8 rRNA genes. The complete genome GC content was 38.9%. In order to reveal the phylogenetic position of *G. verticillata* with other members of Bambusodae, a phylogenetic analysis was performed based on 20 complete chloroplast genomes of Bambusodae, and three taxa (*Acidosasa purpurea, Indosasa sinica, Pleioblastus maculatus*) as outgroups. All were downloaded from NCBI GenBank. The sequences were aligned by MAFFT v7.307 (Katoh and Standley 2013), and the phylogenetic tree was constructed using RAxML (Stamatakis 2014). The phylogenetic tree showed that *G. verticillata* was most closely related to *D. latiflorus* with strong support (Figure 1).

**Figure 1.**
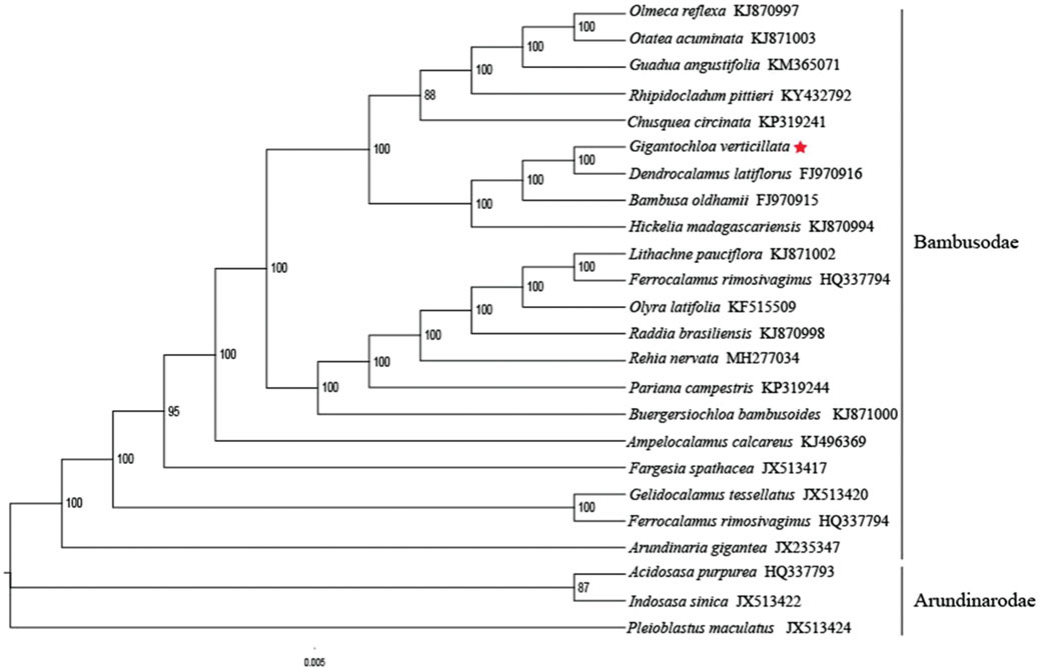
Phylogenetic analysis of 20 species of Bambusodae and three taxa (*Acidosasa purpurea, Indosasa sinica, Pleioblastus maculatus*) as outgroup based on plastid genome sequences by RAxML, bootstrap support value near the branch.
